# Effectiveness of the TEI Program for Bullying and Cyberbullying Reduction and School Climate Improvement

**DOI:** 10.3390/ijerph16040580

**Published:** 2019-02-16

**Authors:** Rosario Ferrer-Cascales, Natalia Albaladejo-Blázquez, Miriam Sánchez-SanSegundo, Irene Portilla-Tamarit, Oriol Lordan, Nicolás Ruiz-Robledillo

**Affiliations:** 1 Department of Health Psychology, Faculty of Health Science, University of Alicante, 03690 Alicante, Spain; rosario.ferrer@ua.es (R.F.-C.); miriam.sanchez@ua.es (M.S.-S.S.); irene.portilla@ua.es (I.P.-T.); nicolas.ruiz@ua.es (N.R.-R.); 2 Management Department, Universitat Politècnica de Catalunya, 08222 Terrassa, Spain; oriol.lordan@upc.edu

**Keywords:** bullying, cyberbullying, school climate, intervention program

## Abstract

The increase in the prevalence of bullying and cyberbullying in recent years worldwide is undeniable. Although several intervention programs oriented towards the reduction of bullying and cyberbullying have been developed and implemented, significant disparities have been found regarding their efficacy. In most of the cases, the lack of the implementation of interventions involving all of the school community could be on the basis of this limited efficacy. The present study aimed to evaluate the effectiveness of the TEI Program, an intervention based on peer tutoring, in the reduction of bullying and cyberbullying, and in the improvement of school climate. The design of the study was quasi-experimental, in which 2057 Spanish students (aged 11 to 16 years) participated from 22 schools, and were randomly assigned to the experimental group (10 schools, 987 students) or the control group (12 schools, 1070 students). The obtained results showed a significant reduction in bullying behavior, peer victimization, fighting, cyberbullying and cybervictimization in the experimental group after the intervention implementation. Similarly, a significant improvement in factors of school climate was found only in this group. The obtained results demonstrated that the TEI program is effective in reducing bully and cyberbully behavior, and at the same time, improving the school climate.

## 1. Introduction

Bullying and cyberbullying, as phenomena increasing in prevalence, are public health problems which entail severely negative consequences for health and quality of life, both for victims and perpetrators [1]. Researchers have found that between 40% and 55% of students in school are involved in some way of victimization (as victims, aggressors or observers), between 20% and 50% report experiences of verbal harassment and between 2% to 7% of students have been victims of a severe form of physical aggression [1,2]. Prevalence rates of bullying behaviors found in different countries vary widely depending on the ages of samples and the period of time over which information is requested [3]. For example, several studies have demonstrated that in Chinese societies school bullying behaviors varies widely according to samples of adolescents aged 10 to 18, which rank from 8% to 52% [4]. For cyberbullying, there are also variations in prevalence rates of self-reported victimization with rates of 55% in North America and Asia, 25% in Canada and 30% in Europe [3,5,6].

The global increase in prevalence in bullying and cyberbullying has supposed the basis of the development of a series of anti-bullying interventions, although some of them possess limited efficacy [7]. A recent meta-analysis has demonstrated that anti-bullying programs effectively reduce school-bullying perpetration by approximately 19–20%, and school-bullying victimization by approximately 15–16% [8]. Among the most significant protective factors against bullying and cyberbullying victimization, school climate, school safety and peer influence have been found to lower rates of victimization among peers [5]. School climate can be defined as the quality of the interactions between students, teachers, parents, and school staff, reflecting the norms, values, and goals that represent the educational and social missions of the school [9]. School and the relationships that adolescents establish in this context play an important role in socioemotional development during adolescence [10]. Some authors indicate that the promotion of safe schools through the improvement of school climate and those in which teachers, students and families are involved, with an active participation of the whole community should be part of the intervention programs oriented to the prevention and intervention of bullying [11,12]. However, these multicomponent programs have been less studied and the results to date are controversial [13]. Thus, while some studies have demonstrated positive results [12], other studies have failed to find a significant association. For example, in a metanalysis by Ttofi and Farrington [13], school-based interventions including work with peers did not demonstrate effectiveness in bullying reduction. The dichotomization of the analyzed variables and the lack of an exhaustive analysis of peer tutoring activities employed in the revised studies could explain these negative results [14]. Moreover, it is likely that this ambiguity in the obtained results is based on the specific characteristics of activities of peer tutoring programs, rather than the nature of the intervention. Given these controversial results, new studies are required to simultaneously identify the variables that could modulate this effectiveness, in which the school climate could play a main role [15].

In this study, we examined the effectiveness of the TEI program, a Spanish initiative for bullying and cyberbullying reduction based on the ecological model of peer tutoring in schools.

### TEI Program “Peer Tutoring”

TEI program, acronyms that refer to the terms in Spanish “*Tutoría Entre Iguales*”, is a school-based intervention of peer-tutoring, oriented towards the prevention of school violence and cyberbullying, and designed for students of secondary education schooling. The main objective of this program is the improvement of the school climate and the promotion of a positive school coexistence through the development of adequate solving problem strategies and the integration of a culture of zero tolerance for violence as an identity school trait. The TEI program is based on an institutional intervention that entails the collaboration and commitment of the whole school community. It is designed on the basis of the Ecological Systems Theory of Bronfenbrenner [16], the principles of emotional intelligence theories from the studies of Salovey and Mayer [17] and Goleman [18]; and positive psychology [19]. The development and implementation of the intervention can be described as follows:

Stage 1: Dissemination and Awareness about the Intervention along the School Community

The beginning of the intervention program is based on the information about and dissemination of the principles of the program between all members of the school community (teachers, families and students). In this stage, the application of the intervention program is approved by the management team of the teaching center. During this stage, families receive information regarding the objectives and characteristics of the program by the TEI staff, a group of specialized education professionals involved in the development and implementation of TEI program in schools. Besides being informed, families are encouraged to be actively involved in the implementation of the program during the school year. Volunteer parents receive training on detection and action against harassment and victimization.

Stage 2: Teacher Training

The TEI staff develops an initial intensive training for teachers with a duration of 30 h (10 h in face-to-face format and 20 h in virtual format). During this educational training, teachers of the school create a TEI group of teachers and a coordinator is designated. This group is responsible for coordinating the implementation of the intervention in the school.

Stage 3: Student Tutors Training

Students tutors receive an initial training of 3 sessions lasting 1 h, based on the socioaffective method. The contents of this training are addressed to personal qualities of tutors, tutor functions, social abilities, prosocial behavior, empathy and problem-solving strategies. The TEI staff carry out 1 session of 1 h quarterly to follow up on the implementation of the intervention with student tutors. About 94% of students from all centers involved in the project were interested in participating as tutors. The rest of the students participated in the coordination by helping the teachers of the school involved in the program. The goal was that all students were involved in some way in the program.

Stage 4: Pairing Students

During this phase, the group of teachers who coordinate TEI implementation in the school created pairs of tutor-tutee, taking into account the age of the participants and the interpersonal skills. The maximus age difference between tutor and tutee was 2 years. Concerning to interpersonal skills, the students were classified by teachers on a likert scale of 1 to 3 (low, medium and high demand) points depending on the vulnerability or risk of harassment and the skills to help their peers. Students with high interpersonal skills were assigned as tutors of vulnerable younger students. Furthermore, an interview between tutors and tutees was conducted, and group dynamics during this session were developed to promote cooperativeness between them.

Stage 5: Intervention Development

This stage is based on the permanent training of the conformed pairs throughout three specific types of activities:

- Cohesion activities. The objective is to consolidate and facilitate the tutor-tutee relationship. 2 sessions are held per quarter during school hours.
- Tutorial activities. A formal tutoring is carried out each month between the tutor and tutee, as well as between tutors and program coordinators. In the same way, there are also informal tutorials that take place during recess, when leaving class, in the corridors, etc. The development of informal tutorials is highly promoted in the intervention program, taking into account that they are considered the main key to achieve the objectives of the intervention. The student tutor keeps a record of the informal tutorials carried out.
- Specific training activities. The program includes an intervention of 9 sessions lasting 1 h each, distributed throughout the academic year within the tutorial action plan. Each session is aimed towards the development of a specific skill. The methodology of the sessions is as follows: tutors present the skill to be worked and the specific activity to perform in class. Then, the activity is put into practice through specific tasks, and finally, tutors and tutees summarize the contents of the session by completing a mural or graphic poster in order to transfer the knowledge worked in the session to the whole school. The contents of the sessions are: emotional self-knowledge, emotional regulation, social competences and the positive use of ICTs.

Stage 6: Closing

A joint activity aimed to the diploma delivery accrediting participation in the TEI program to all involved agents (tutors, tutees, teaching staff and family volunteers) is performed at the end of the academic year.

Bearing all this in mind, the main aim of the present study is to evaluate the effectiveness of the TEI program reducing bullying and cyberbullying and promoting a positive school climate in schools. Following the results of the previous research regarding this issue, it has been hypothesized that the TEI program, based on peer tutoring, will be effective in the reduction of bullying and cyberbullying [12]. In the same way, it is expected that the application of this program in schools results in a school climate improvement [12].

## 2. Materials and Methods

### 2.1. Procedure

The sample was derived from a randomized small-scale trial of peer tutoring program in 22 public secondary education schools of Spain. The program and the battery of instruments used were presented and explained to the centers’ management teams, who valued it positively. They presented it to each School Council as part of the Coexistence Project and Improvement Plan of the Educational Center who granted informed consent to participate in it. Secondary education schools were matched at baseline based on size and demographics to condition (i.e., intervention vs wait-list control). All procedures performed in the present study were in accordance with the ethical standards of the University of Alicante and the Educational Directive Committee from Schools involved in the study (Ref number: UA2015-1013).

The final sample included 2389 students who enrolled in the project in the fall of 2015. The inclusion criteria were: (1) presence in the classroom on the day of the survey; (2) ability to read and complete the questionnaires; (3) informed consent of parents and adolescents over 12 years of age before data collection. In total, 2057 students were included in the final analyses, since they had participated and completed phases T1 and T2 (86.1% of the initial sample) and their questionnaires could be correctly matched. The abandonment of just over three hundred and thirty-two students was mainly due to errors when completing the corresponding identification code that the students themselves had to create to guarantee their anonymity or absence on the day of data collection.

Students data collection was conducted in September-October 2015 (baseline, T1) using online surveys for the Experimental Group (EG) and Control Group (CG). Only students, teachers and families of the EG received the intervention. The second data collection (follow-up T2), took place after seven months, May-June 2016, a few weeks before the end of the same school year. Data for the CG were collected during the same period as for the EG.

### 2.2. Participants

Participants included 2057 students, of which 49.6 % (*n* = 1021) were female and 50.4 % (*n* = 1036) male. Participants ranged in age from 11 to 16 years at the beginning of the program, with a mean age of 13.08 (SD = 1.18). 987 students from 10 secondary education schools were assigned to the experimental group. A total of 496 students of third year of secondary education were tutors of 491 first year tutees; and 1070 students from 12 schools were assigned the control group. There were no significant differences between groups in gender (X^2^ = 1.104, *p* = 0.29). However, in age, differences between groups were found (*t* = –2.962, *p* = 0.001). For that reason, age was controlled in the subsequent analyses as a covariate.

### 2.3. Measures

#### 2.3.1. Bullying

The Illinois Bully Scale (20) is an 18 item, self-report measure that contains three subscales for measuring the frequency of fighting, peer victimization, and bully behavior. Students were asked to indicate how often in the past 30 days they have engaged in each behavior. Response options included “Never,” “1 or 2 times,” “3 or 4 times,” “5 or 6 times,” and “7 or more times.” These response options allow the assessment of the persistence of the bullying. Higher scores indicate more self-reported bullying behaviors. The Spanish version of the Illinois Bully Scale in the present study was found to be a reliable and valid measure of bullying behavior showing a factor structure that fit indices in Spanish adolescents with a Kaiser-Meyer-Olkin index and Barlett sfericity (KMO = 0.906; χ^2^ = 16,994.404; gl = 153; *p* = 0.00) explaining the 58.01% of variance. While the confirmatory factor analysis obtains optimal adjustment indices χ^2^ = 521.377; RMSEA = 0.041; SRMR = 0.029; CFI = 0.976; GFI = 0.973; NNFI = 0.968; and RFI = 0.959 according to Hu and Bentler [20]. Internal consistency (Cronbach’s alpha) for each factor in the sample analyzed in this study was good: bully behavior (α = 0.89); peer victimization (α = 0.75) and frequency of fighting (α = 0.76).

#### 2.3.2. Cyberbullying

Cyberbullying was evaluated employing the E-Victimization Scale (E-VS) and E-Bullying Scale (E-BS) [21]. These scales identify bullies and victims of bullying that use electronic devices and ICTs developed for youth. The E-VS includes 5 items, while the E-BS is composed of 6 items, all of them introduced by the sentence: “In the last 7 days…” Students are asked to indicate the frequency (from 0 to 6 or more times) of which they have suffered (E-VS) or have inflicted (E-BS) certain behaviors via ICTs. Given this scale were not available for the wider Spanish population, we followed the international standards published by the International Test Commission, using the translation-back-translation process [22,23]. In the validation these scales show their suitability in the exploratory factor analysis with a Kaiser-Meyer-Olkin index and Barlett sfericity (KMO = 0.856; χ^2^ = 9960.413; gl = 55; *p* = 0.001) explaining the 58.65% of variance. The confirmatory factor analysis obtains optimal adjustment indices χ^2^ = 169.689; RMSEA = 0.039; SRMR = 0.024; CFI = 0.986; GFI = 0.985; NNFI = 0.981; and RFI = 0.975 according to Hu and Bentler [24]. Internal consistency (Cronbach’s alpha) for each factor in the analyzed sample in this study was good: E-VS (α = 0.85) and E-BS (α = 0.80).

#### 2.3.3. School Climate

The Spanish version of the school climate questionnaire has been previously validated for evaluating the quality of school climate in Spanish high-schools, showing adequate psychometric properties in this context [25]. The overall instrument is composed of 49 items, grouped into 10 subscales ranked on a 4-point Likert scale. In the present study we only included four subscales assessing Satisfaction with school (8 items), Sense of belonging (5 items), Cooperation (3 items), and Communication between family and school (6 items). These subscales have showed good reliability coefficients, with alpha Cronbach values ranking from α = 0.88 for Satisfaction; α = 0.75 for Sense of belonging; α = 0.76 for Cooperation; and α = 0.85 for Communication between family and school.

### 2.4. Data Analysis

For the analyses, scores of participants from the EG and CG in each of the evaluated variables were employed. First, T-test comparison between EG and CG were employed to analyse possible differences in baseline between groups. Effect size estimation was also computed for each pair of variables using Cohen’s definitions (1988). ANCOVA of repeated measures of ‘moment’ (T1 pre-intervention vs T2 post-intervention) with ‘group’ (participants from the EG vs participants from the CG) as between-subject factor was performed to analyze the effectiveness of the intervention reducing bullying and cyberbullying, and improving school climate. For significant results, partial eta-squared (*η*^2^) was reported as a measure of the effect size [26]. Furthermore, the percentage change between scores from T1 to T2 was calculated following this formula: [(T2 − T1/T1)]*100. As has been previously indicated, age was introduced in the analyses as a covariate. All statistical analyses were performed using SPSS (International Business Machines Corporation (IBM), Armonk, NY, USA), Statistics for Windows, Version 23.0, considering *p* < 0.05 to be significant. The descriptive values are expressed as mean and standard deviation (M and SD, respectively).

## 3. Results

### 3.1. Descriptive Statistics in T1 and T2 for the Both Groups (EG and CG) and Percentage Change for Bullying, Cyberbullying and School Climate

Regarding bullying and cyberbullying, the conducted analyses did not revealed significant differences between the EG and the CG in baseline scores (T1) for bully behavior (*t* = 1.57, *p* = 0.11; Cohen’s *d* = 0.06), peer victimization (*t* = −0.55, *p* = 0.57; Cohen’s *d*= 0.02), fighting (t = 0.68, *p* = 0.49; Cohen’s *d* = 0.03), cyberbullying (*t* = 1.40, *p* = 0.16; Cohen’s *d* = 0.06) or cybervictimization (*t* = 1.11, *p* = 0.26; Cohen’s *d* = 0.04). With regard to school climate, no differences were found between groups in satisfaction (*t* = −1.21, *p* = 0.22; Cohen’s *d* = −0.05), sense of belonging (*t* = 1.41, *p* = 0.15; Cohen’s *d* = 0.06), cooperation (*t* = −1.33, *p* = 0.18; Cohen’s *d* = −0.05) and communication between family and school (*t* = −1.12, *p* = 0.25; Cohen’s *d* = − 0.04), measured at baseline (Table 1).

The results regarding the impact of the TEI program on the prevalence of the different variables showed different percentage variations in CG and EG. Table 1 shows how the involvement in bully/victimization and cyberbullying/cybervictimization decreased over time only in EG; while the school climate subscales mostly increased in the students who participated in the peer tutoring program.

### 3.2. Effectiveness of the Intervention in Bullying Reduction

**Figure 1:**
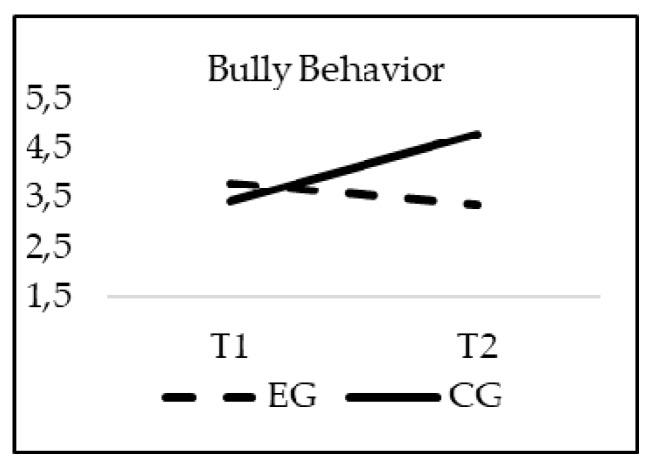
Interaction effects of group (EG-CG) and time (T1-T2) in Bully Behavior.

**Figure 2:**
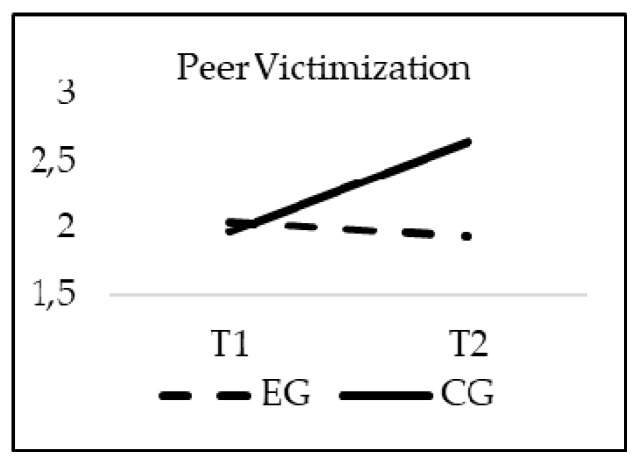
Interaction effects of group (EG-CG) and time (T1-T2) in Peer Victimization.

**Figure 3:**
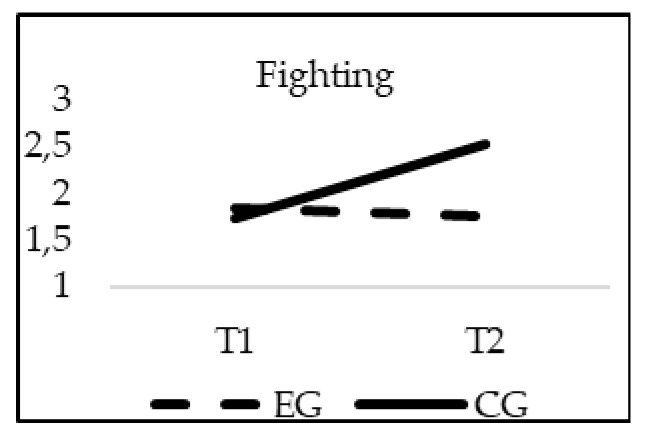
Interaction effects of group (EG-CG) and time (T1-T2) in Fighting.

The obtained results show a significant effect of the interaction group*time for Bully behavior (F_(1, 20)_ = 30.973; *p* = 0.00; *η*^2^ = 0.015), Peer victimization (F_(1, 20)_ = 15.299; *p* = 0.00; *η*^2^ = 0.007) and Fighting (F_(1, 20)_ = 19.552; *p* = 0.00; *η*^2^ = 0.009). As previously indicated, age was introduced in the model as a covariate, but did not reach statistical significance for Bully behaviors (F_(1, 20)_ = 9.389; *p* = 0.256), Peer victimization (F_(1, 20)_ = 0.2; *p* = 0.644), Fighting (F_(1, 20)_ = 0.318; *p* = 0.573). The values of the Bully behavior, Peer victimization and Fighting subscales decreased significantly from T1 to T2 only in the students who participated in the TEI intervention program, finding statistically significant differences between the EG and CG in T2 (*p* = 0.001) (see Figure 1, Figure 2 and Figure 3).

### 3.3. Effectiveness of the Intervention in Cyberbullying Reduction

**Figure 4:**
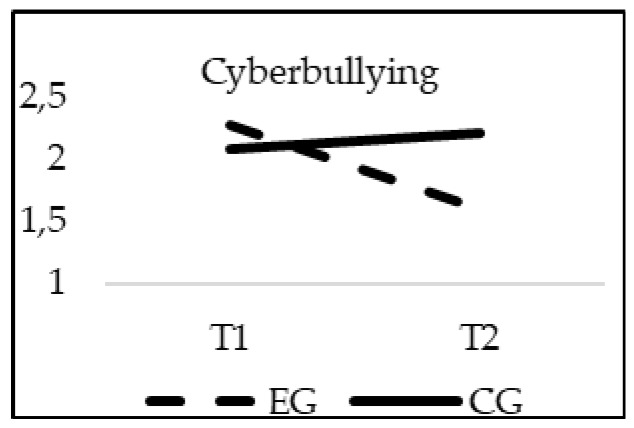
Interaction effects of group (EG-CG) and time (T1-T2) in Cyberbullying (E-Bullying Scale).

**Figure 5:**
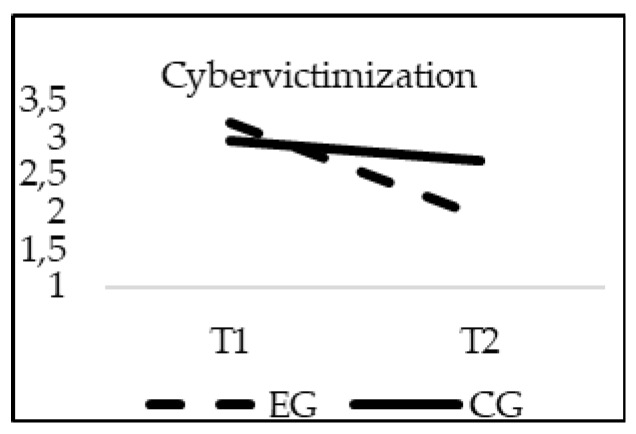
Interaction effects of group (EG-CG) and time (T1-T2) in Cybervictimization (E-Victimization Scale).

With respect to Cyberbullying, a significant interaction effect of group*time was found for Cyberbullying (E-Bullying Scale) (F_(1, 20)_ = 12.382; *p* = 0.000; *η*^2^ = 0.006), and Cybervictimization (E-Victimization Scale) (F_(1, 20)_ = 9.516; *p* = 0.002; *η*^2^ = 0.005). When age was introduced in the model as a covariate, no significant statistical effects of this variable were found for Cyberbullying (F_(1, 20)_ = 2.733; *p* = 0.098) or Cybervictimization (F_(1, 20)_ = 0.435; *p* = 0.510). As it can be seen in Figure 4 and Figure 5, in the CG the scores were similar from T1 to T2 (*p* > 0.05). However, in the EG the scores were significantly lower in the T2 phase, finding statistically significant differences between the EG and the CG in T2 (*p* = 0.001).

### 3.4. Effectiveness of the Intervention in School Climate Improvement

**Figure 6:**
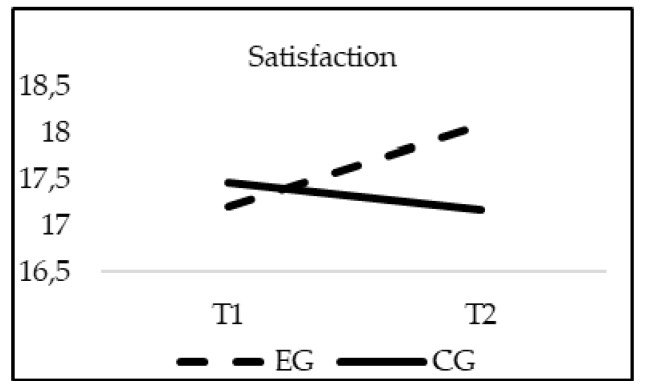
Interaction effects of group (EG-CG) and time (T1-T2) in Satisfaction.

**Figure 7:**
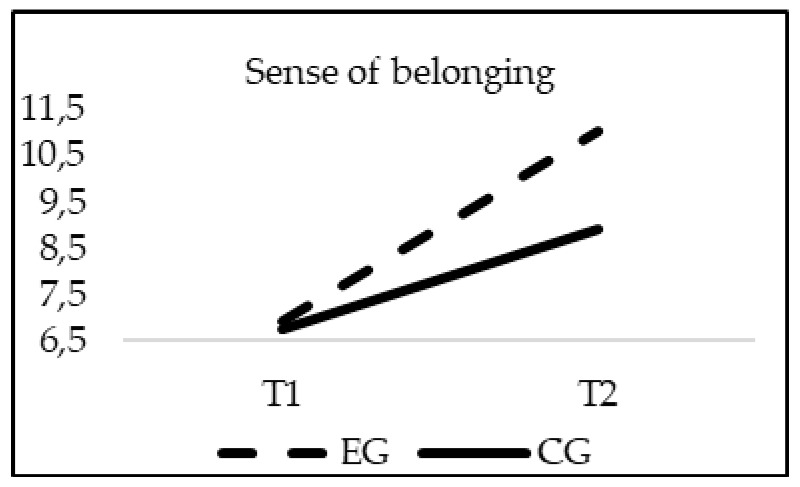
Interaction effects of group (EG-CG) and time (T1-T2) in Sense of belonging.

**Figure 8:**
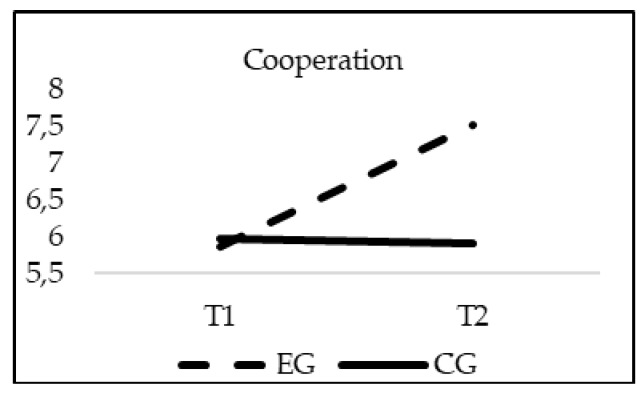
Interaction effects of group (EG-CG) and time (T1-T2) Cooperation.

**Figure 9:**
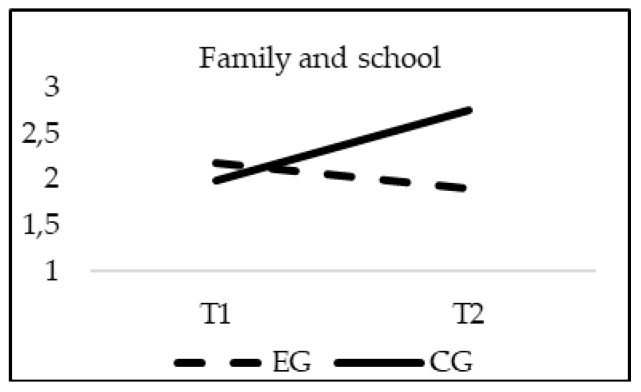
Interaction effects of group (EG-CG) and time (T1-T2) in Communication between family and school.

The analyses of differences regarding School Climate showed a significant effect of the interaction group*time in Satisfaction (F_(1, 20)_ = 16.818; *p* = 0.000; *η*^2^ = 0.008); Sense of belonging (F_(1, 20)_ = 126.234; *p* = 0.00; *η*^2^ = 0.058); Cooperation (F_(1, 20)_ = 195.768; *p* = 0.00; *η*^2^ = 0.089) and Communication between family and school (F_(1, 20)_ = 233.528; *p* = 0.00; *η*^2^ = 0.102). As in the previous cases, when age was introduced in the model as a covariate, this variable did not reach statistical significance in any of the variables of the School Climate: Satisfaction (F_(1, 20)_ = 2.056; *p* = 0.152), Sense of belonging (F_(1, 20)_ = 2.515; *p* = 0.113), Cooperation (F_(1, 20)_ = 2.132; *p* = 0.4), and Communication between family and school (F_(1, 20)_ = 1.853; *p* = 0.174). Although the EG and CG obtained similar scores in all of the subscales of the School Climate questionnaire in T1 (*p* > 0.05), only the experimental group significantly increased their scores in all variables of school climate in T2, finding statistically significant differences between groups at this moment (*p* = 0.001) (Figure 6, Figure 7, Figure 8 and Figure 9).

## 4. Discussion

The present study aimed at the evaluation the effectiveness of the TEI Program in the reduction of bullying and cyberbullying, and the improvement of school climate in a sample of adolescents. Our results suggest that the Spanish TEI program may be effective for improving school climate factors including satisfaction with the school, sense of belonging, cooperation and positive communication between family and school. These factors were positively related to low rates of fighting, bullying and cyberbullying victimization.

The TEI program is based on its highly organized proposal, the involvement of all members of the educational community in the intervention and the development of a set of specific materials and activities that could be adapted to the school needs and context. Different training activities are carried out, paying special attention to the formal and informal tutorials that the students will participate in with other students and with the coordinator. In this sense, as previously indicated, the program puts a lot of emphasis on informal tutorials, which are considered as the key element to achieve the objectives of the intervention by generating a real and informal support network between students. These actions generate an environment where cooperation, communication and negotiation are highly promoted, giving voice and space to the students and to the whole educational community. Thanks to this, members of the school community could manifest openly their personal needs, creating situations of social learning in the school environment. In this context, students listen to each other, detect possible situations of interpersonal conflict or harassment and empathize with peers in difficult situations. In this way, students are empowered in order to develop a feeling of coexistence in which violence has no place.

Contrary to previous research in which interventions based on peer tutoring did not demonstrate effectiveness in the reduction of bullying among schools [13], this research pointed out how programs created following this theoretical approach could be useful for this aim. As has been explained by Cowie and others [27,28], peer-tutoring could be an excellent tool for violence reduction in schools by promoting an increase of self-perception and introspection abilities in students, while interpersonal, social and conflict resolution skills are trained. The increase of befriending, mediation skills and active listening have been proposed as the main mechanisms that could be on the basis of violence reduction in schools communities which implement peer-tutoring interventions [27]. This fact entails the development of a caring school community in which violence has no place. Unlike other type of programs in which the intervention of teachers is more directive and invasive, peer-tutoring gives responsibility to students, an approach that ethological studies of conflict resolutions have demonstrated to be the best strategy [27]. Furthermore, this intervention approach has benefits for the whole community, but fundamentally, for tutors and tutees. In a classic study aimed to identify positive results of peer tutoring both for tutors and tutees, the author identified that after the peer tutoring intervention, tutors had more self-confidence, developed a higher grade of responsibility and experienced an increase of their feelings of belonging to the school community [29]. Furthermore, peer tutoring usually improved their self-esteem, as they assumed the responsibility of doing something that contributed to the improvement of their school environment. The reasons that they indicated for these changes were based on the interpersonal and prosocial behavior training that they received, mainly active listening. For tutees and possible victims, the program gives them the possibility of communicating what happens to them or having favorable interlocutors to transmit it, since tutors are peers that are willing to help and are usually in a better social position in the school [30]. Aggressors are also influenced by the intervention, because their performance and the environment generated by peer tutoring forces them to perceive, evaluate and become aware about the acts they carry out and the negative consequences of harming their peers. Witnesses of bullying are also affected, so the capacity of reflection regarding violence is fostered in the program, and they are more aware of every form of abuse, even those may be more hidden or unnoticed, adopting a more active behavior in situations of bullying and cyberbullying [30].

All of these results derived from peer tutoring interventions are clearly opposed to the development and maintenance of school violence, so they could be proposed as possible mechanisms explaining the effectiveness of the TEI program in violence reduction. Therefore, the development of prosocial and altruistic behaviors, the instruction regarding problems solving strategies such as active listening and the promotion of empathy towards peers could be on the basis of the effectiveness of peer tutoring programs. However, this type of interventions is oriented towards the whole school community, and for that reason, other complementary explanations should be addressed, putting the focus on global variables, such as school climate. The improvement of school climate could be one of the underlying mechanisms of the positive obtained results regarding the efficacy of TEI program on bullying and cyberbullying. In this sense, significant improvements have been found in all of the variables that characterized this construct. Probably, one of the main mechanisms explaining the effectiveness of the proposed intervention program could be based on the demonstrated improvement of the school climate in the experimental group. Recent research has demonstrated that school climate has direct impacts on bullying. A school in which student feels safe, comfortable and engaged with the community will probably show lower rates of bullying, as demonstrated in previous research [15]. In our study, the students who received the intervention exhibited significant improvements in general satisfaction with the school, as a significant increase of engagement feelings, cooperation between students and communication between families and the school. It is likely that the intervention program could promote a better school climate characterized by the promotion of better school connectedness. Defined as the sense of belonging, attachment, and bonding, a school climate in which students receive positive social support from their peers and teachers increases their academic engagement and could reduce their risk of violent behaviors, such as bullying [15]. The perception of school connection and caring relationships with peers, teachers and school staff, feeling socially connected and supported, could prevent the phenomenon of peer violence in school.

Although the present study entails a significant advance in the comprehension of the effectiveness of anti-bullying interventions based on peer-tutoring in the bullying and cyberbullying reduction, some limitations of the research should be pointed out. First, the use of self-report measures, with the inherent bias of social desirability, is a limitation of the study. Future studies should include hetero-report measures in which parents and teachers discuss adolescent attitudes and behaviors and/or observational techniques to assess and ratify the program’s effects. Second, the procedure of the study covered only two assessment times, pre and post-intervention. Due to the controversial results obtained in previous research regarding the long-term effects of school-based interventions for bullying reduction, it would be recommendable to have a longitudinal follow up after the end of the program. This fact will be addressed by future studies, throughout the evaluation of the long-term effectiveness of the TEI program. Moreover, participants of the present study are students from a specific location in Spain, which could be a limitation for the generalization of the obtained results. Future studies should address this issue by replicating the present study internationally. In any case, despite the aforementioned limitations, the design of the study was quasi-experimental, with a randomized sample, which has been identified as the best procedure to explore the effectiveness of school-based interventions. Furthermore, the sample is large in both groups, and the main types of school violence have been evaluated, bullying and cyberbullying, demonstrating the effectiveness of TEI program for the reduction of both, and for the improvement of school climate.

## 5. Conclusions

The peer tutoring program analyzed in this research has an institutional character that involves the entire educational community, generating a creation of a culture of peaceful coexistence and non-violence in schools, fostering greater emotional education and more satisfactory relations between peers. In this way, the prevention, detection and intervention of situations of violence between peers by students is highly promoted, as is the main objective of the intervention program. Recalling that bullying and cyberbullying are one of the main public health problems affecting adolescents and their families, the necessity of developing and implementing effective interventions in schools is extremely urgent. To our knowledge, this is the first study seeking to analyze the effectiveness of TEI program reducing bullying and cyberbullying and improving school climate among Spanish students. In this regard, the present research demonstrates that the TEI program is an effective and useful intervention program to be applied in schools, in order to prevent and reduce school violence. This fact highlights the importance of implementing interventions based on peer tutoring programs, as its efficacy has been widely demonstrated in previous research [14,31,32]. In this sense, the TEI program could be a useful tool for educators and clinicians who work in school environments and for professionals in need of validated and efficient intervention strategies for the reduction of all forms of school violence.

## Figures and Tables

**Table 1:** Mean and Standard Deviation (T1-T2) and percentage change by groups.

Variable	Scale	Condition EG (*n* = 987) CG (*n* = 1070)	Mean (SD) T1	Mean (SD) T2	Percentage Change
Bullying	Bully behavior	EG	3.80 (5.76)	3.37 (4.68)	−11.31%
		CG	3.44 (4.51)	4.80 (5.92)	39.53%
	Peer victimization	EG	2.05 (2.93)	1.93 (2.84)	−5.85%
		CG	1.97 (2.94)	2.64 (3.50)	34.01%
	Fighting	EG	1.83 (2.89)	1.76 (2.84)	−3.82%
		CG	1.74 (2.81)	2.52 (3.62)	44.82%
Cyberbullying	Cyberbullying	EG	2.27 (3.10)	1.59 (3.80)	−29.95%
		CG	2.08 (3.13)	2.21 (4.48)	6.25%
	Cyber victimization	EG	3.19 (4.82)	1.94 (4.51)	−39.18%
		CG	2.95 (5.10)	2.70 (5.30)	−8.47%
School climate	Satisfaction	EG	17.20 (4.65)	18.08 (4.78)	5.11%
		CG	17.46 (4.98)	17.17 (4.68)	−1.66%
	Sense of belonging	EG	6.92 (2.50)	11 (2.82)	58.95%
		CG	6.76 (2.60)	8.94 (2.98)	32.24%
	Cooperation	EG	5.86 (2.)	7.51 (1.61)	28.32%
		CG	5.98 (1.99)	5.91 (2.06)	−1.66%
	Communication between family and school	EG	13.65 (3.47)	15.98 (2.45)	17.06%
		CG	13.82 (3.62)	13.12 (3.29)	−5.06%
